# Sestrin2 is involved in asthma: a case–control study

**DOI:** 10.1186/s13223-019-0360-3

**Published:** 2019-08-14

**Authors:** Yanfang Kang, Chen Chen, Xiaotian Hu, Xiaohua Du, Huifen Zhai, Yan Fang, Xiulin Ye, Weimin Yang, Shibo Sun

**Affiliations:** 1 Department of Respiratory and Critical Care Medicine, First Affiliated Hospital Kunming Medical University, No.295, Xichang Road, Wuhua District, Kunming, China; 2 2015 Innovation Class, Kunming Medical University, Kunming, China; 3 School of Pharmaceutical Science & Yunnan Key Laboratory of Pharmacology for Natural Products, Kunming Medical University, Kunming, China; 4 Department of Respiratory Medicine, Beijing Friendship Hospital, Capital Medical University, Beijing, China

**Keywords:** Sestrin2, Asthma, Oxidative stress, Inflammation

## Abstract

**Background:**

Asthma is a chronic disease that seriously harms the health of patients. Oxidative stress is involved in asthma. As an oxidative stress-inducible protein, sestrin2 is elevated in oxidative stress-related diseases. We aimed to explore whether sestrin2 was involved in asthma.

**Methods:**

Seventy-six subjects (44 in the asthma group, 32 in the control group) were recruited in this study. Plasma sestrin2 levels, peak expiratory flow (PEF), forced expiratory volume in 1 s (FEV_1_) % predicted, forced vital capacity (FVC) % predicted and FEV_1_/FVC ratio were measured in controls and in asthmatics both during an exacerbation and when controlled after the exacerbation.

**Results:**

The asthma group had a significant higher sestrin2 level than the control group (asthmatics during exacerbation, 1.75 ± 0.53 ng/mL vs. 1.32 ± 0.48 ng/mL, p < 0.001; asthmatics when controlled after the exacerbation, 1.56 ± 0.46 ng/mL vs. 1.32 ± 0.48 ng/mL, p = 0.021, respectively). In addition, sestrin2 was negatively correlated with FEV_1_% predicted and FEV_1_/FVC ratio in asthmatics during exacerbation (r = − 0.393, p = 0.008; r = − 0.379, p = 0.011; respectively). Moreover, negative correlations between sestrin2 and FEV_1_% predicted and FEV_1_/FVC ratio also existed in asthmatics when controlled after the exacerbation (r = − 0.543, p < 0.001; r = − 0.433, p = 0.003 respectively). More importantly, multiple linear regression analysis demonstrated that FEV_1_% predicted was independently associated with sestrin2 in asthmatics both during exacerbation and when controlled after the exacerbation.

**Conclusions:**

Sestrin2 is involved in asthma. Sestrin2 levels increase in asthmatics both during exacerbation and when controlled after the exacerbation. In addition, sestrin2 is independently associated with FEV_1_% predicted.

## Background

Asthma is a common disease clinically characterized by recurrent wheeze, shortness of breath, chest tightness and cough [1]. Currently, 235–334 million people worldwide suffer from this disease which seriously endangers their health and even causes death [2, 3]. According to WHO, the estimated death due to asthma in 2015 was 383,000 [2]. Asthma is usually characterized by chronic airway inflammation which leads to airway remodeling and fixed airflow limitation followed by poor asthma control [1]. Accordingly, airway inflammation is one of the most important targets of asthma treatment. Currently, the diagnosis and treatment of asthma are usually based on the pattern of symptoms in combination with lung function. However, symptoms and lung function, to some extent, may not fully reflect the level of inflammation [4]. Biomarkers can partly reflect the level of airway inflammation [5]. In addition, biomarkers may provide assistance for diagnosis, targeted treatment and monitoring of asthma [6]. Recently, several biomarkers have been reported, including immunoglobulin E (IgE), eosinophil, neutrophil, periostin, dipeptidyl peptidase 4, protein YKL-40, cluster of differentiation 93 (CD93), etc. [4, 7–9].

Sestrin2, also known as hypoxia-inducible protein 95, is an oxidative stress-inducible protein whose expression is regulated by p53 [10–12]. In mammals, sestrin2 has two biochemical functions. Firstly, as an antioxidant that controls peroxidase activity, sestrin2 scavenges reactive oxygen species (ROS) to reduce oxidative stress [12]. Secondly, in the case of oxidative stress, p53-induced sestrin2 activates AMP-dependent protein kinase (AMPK) and inhibits mammalian target of rapamycin protein to up-regulate autophagy catabolism [11, 13, 14]. In addition, sestrin2 increases in oxidative stress-related diseases such as COPD [15].

It was reported that oxidative stress increased in asthma and caused airway inflammation and airway remodeling [16, 17]. Therefore, we speculated that sestrin2 might also be involved in asthma. The aim of this study was to explore whether sestrin2 was involved in asthma to set the stage for future study.

## Methods

### Subjects

All 76 subjects were recruited from the First Affiliated Hospital of Kunming Medical University. 44 patients were from the outpatient service or hospital admission for asthma exacerbation treatment, and 32 healthy subjects were from the Health Examination Center of hospital. Comprehensive physical examination, laboratory test and medical history collection were performed for all patients prior to enrollment.

Inclusion criteria: (1) Patients visited a doctor for asthma exacerbation; Exacerbations of asthma are episodes characterized by progressive increase in symptoms of shortness of breath, cough, wheezing or chest tightness and progressive decrease in lung function, which means the current treatment needs to be changed to meet the patient’s requirements according to the Global Strategy for Asthma Management and Prevention (GINA) guidelines [1]. (2) Patients who had been diagnosed with asthma prior to enrollment according to clinical history of symptoms with airflow limitation and positive bronchial provocation or bronchodilatation test. Positive bronchodilatation was defined that the FEV_1_ increased > 12% and 200 ml from baseline, 10–15 min after 200–400 mcg albuterol being inhaled, and positive provocation was defined that FEV_1_ decreased by 15% of baseline with hypertonic saline challenge according to the GINA guidelines [1]. (3) The lung function data [peak expiratory flow (PEF), forced expiratory volume in 1 s (FEV_1_) % predicted, forced vital capacity (FVC) % predicted and FEV_1_/FVC ratio] were available at exacerbation and relief from exacerbation with treatment.

Exclusion criteria: (1) Patients who suffered from lung diseases other than asthma; (2) Patients with cardiovascular system diseases, kidney disease, or metabolic diseases, etc.; (3) Pregnant patients.

This study was approved by the Ethics Committee of Hospital, and informed consent was obtained before the study.

### Treatment of asthma exacerbation

The treatments were divided into three levels. First level: patients were treated with inhaled salbutamol or budesonide/formoterol at the time of first arrival to the outpatient service or admission. Second level: the efficacy of the first level treatment was evaluated 1–2 h later. If the patient’s symptoms didn’t improve, the doxofylline accompanied with inhaled budesonide and terbutaline were added. Third level: the efficacy of the second level treatment was evaluated 4–6 h later, and oral or intravenous methylprednisolone and montelukast would be used on the basis of second level treatment if the patient’s symptoms still didn’t improve. The patients who couldn’t be improved with treatment of the three levels or patients who needed mechanical ventilation were excluded from this study.

### Relief of asthma exacerbation

At any level of the treatment above, if the patient’s symptoms were improved, the treatment would be maintained until the patient’s symptoms disappeared. Then the treatment of asthma exacerbation was completed and the patients were discharged. The subsequent treatment was changed to inhaled corticosteroids (ICS)/long-acting beta2-agonist (LABA). In the following 4 weeks of discharge, the patients who met with the GINA criteria for well or partly controlled asthma were included in this study [1]. The patients whose asthma was uncontrolled were excluded from this study.

### Blood test

Venous blood samples were obtained at two time spots: first arrival to the outpatient service before treatment or at the time of admission before treatment; at the end of the treatment in hospital. Following a centrifugation at 3000 rpm for 20 min, the supernatant was taken and stored at − 80 °C for testing. Plasma sestrin2 concentrations were detected using an ELISA kit (Mlbio, China). The lung function test was completed by the laboratory of First Affiliated Hospital of Kunming Medical University.

### Statistical analysis

The data were expressed as mean ± standard deviation, and the one-sample Kolmogorov–Smirnova method was used to detect whether the data were normally distributed. Normally distributed data were compared using an unpaired t test. Paired *t* test was used in comparison between before and after treatment, and rank sum test was used for non-normal distribution data. Spearson’s correlation analysis was used to test the correlations between sestrin2 and age, gender, body mass index (BMI), smoking index, PEF, FEV_1_% predicted, FVC% predicted, and FEV_1_/FVC ratio. Multiple linear regression analysis with stepwise selection was used to determine the relationships between sestrin2 and age, BMI, smoking index, FEV_1_% predicted, PEF, FVC% predicted, and FEV_1_/FVC ratio. Statistical analyses were performed with SPSS17.0.

## Results

Seventy-six subjects were enrolled in the study and divided into asthma group (n = 44) and control group (n = 32). The demographic characteristics, results of lung function and sestrin2 test were listed in Table 1.

**Table 1:** Demographic characteristics, lung function tests, sestrin2 results of the asthma and control groups

	Control group (n = 32)	Asthma group (n = 44)	p value
Age (years)	41.66 ± 7.74	42.95 ± 8.47	0.496
Gender, male (n, %)^a^	15 (46.88)	21 (47.73)	0.563
BMI (kg/m^2^)	26.45 ± 3.06	27.26 ± 3.98	0.338
Smoking index (pack year)	3.22 ± 7.91	2.52 ± 6.06	0.665
Treatment before enrollment			
ICS (never/former or current)	–	19/25	–
LABA (never/former or current)	–	28/16	–
SABA (never/former or current)	–	7/37	–
LAMA (never/former or current)	–	21/13	–
Causes of exacerbation			
Allergen exposure	–	9	–
Poor adherence	–	18	–
Respiratory infection	–	13	–
Undistinguishable	–	4	–
PEF (l/min)	318.8 ± 47.91	243.75 ± 43.18	< 0.001*
		311.75 ± 36.11^#^	
FEV1% predicted (%)			
Before treatment	91.57 ± 13.68	67.90 ± 15.66	< 0.001*
After treatment	–	72.35 ± 11.78^#^	–
FVC% predicted (%)			
Before treatment	95.75 ± 11.06	82.94 ± 12.54	< 0.001*
After treatment	–	90.30 ± 10.84^#^	–
FEV1/FVC ratio (%)			
Before treatment	88.80 ± 5.29	77.35 ± 14.54	< 0.001*
After treatment	–	82.98 ± 2.11^#^	–
Sestrin2 (ng/mL)			
Before treatment	1.32 ± 0.48	1.75 ± 0.53	< 0.001*
After treatment	–	1.56 ± 0.46^#^	–
Treatment time in hospital (days)	–	3.64 ± 1.95	–

Data are presented as mean ± SD

*BMI* body mass index, *PEF* peak expiratory flow, *FEV*_*1*_ forced expiratory volume in 1 s, *FVC* forced vital capacity, *ICS* inhaled corticosteroids, *LABA* long-acting beta2-agonist, *SABA* short-acting beta2-agonist, *LAMA* long-acting muscarinic antagonist

* Statistically significant difference

^#^ *P *< 0.05, vs. before treatment

^a^Data are presented as number and rate

**Fig. 1:**
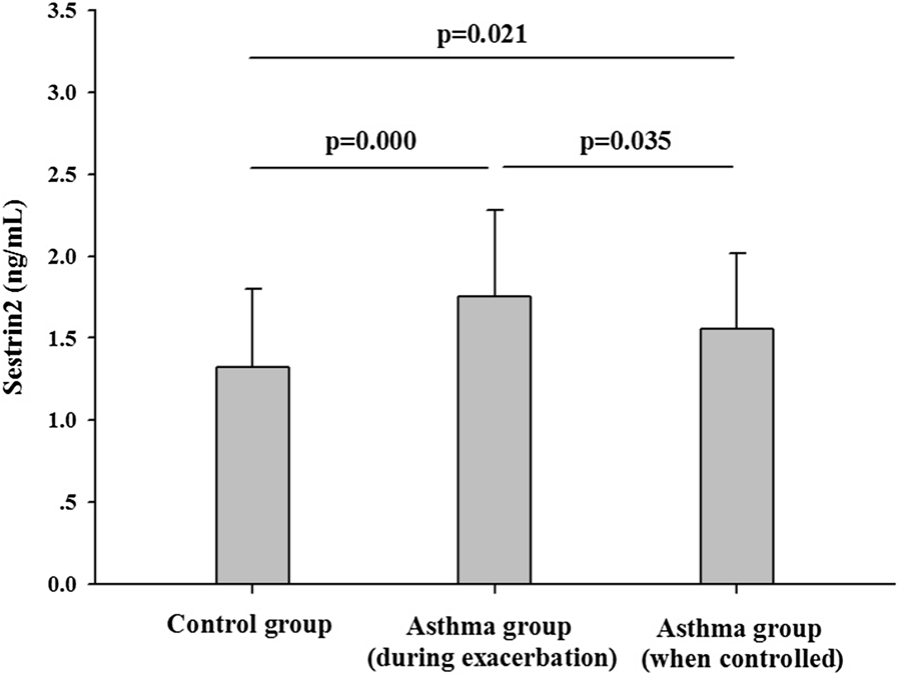
Concentrations of sestrin2 in the asthma and control groups

Comparisons between sestrin2 concentrations in control group and in asthma group (during exacerbation and when controlled after the exacerbation) were presented in Fig. 1. Both during exacerbation and when controlled after the exacerbation, the asthma group had significant higher sestrin2 level than that of the control group (asthma during exacerbation, 1.75 ± 0.53 ng/mL vs. 1.32 ± 0.48 ng/mL, p < 0.001; asthma when controlled after the exacerbation, 1.56 ± 0.46 ng/mL vs. 1.32 ± 0.48 ng/mL, p = 0.021, respectively). Compared with the sestrin2 level in asthmatics during exacerbation, the sestrin2 level decreased in asthmatics when asthma was controlled after the exacerbation (1.75 ± 0.53 ng/mL vs. 1.56 ± 0.46 ng/mL, p = 0.035).

Correlations between sestrin2 and lung function parameters were listed in Table 2. The level of sestrin2 was negatively correlated with PEF, FEV_1_% predicted and FEV_1_/FVC ratio in asthmatics during exacerbation. Moreover, negative correlations between sestrin2 and FEV_1_% predicted and FEV_1_/FVC ratio also existed in asthmatics when asthma was controlled after the exacerbation (Table 2).

**Table 2:** Spearman’s correlations between sestrin2 and the other factors

	Asthma during exacerbation		Asthma when controlled	
	r	p value	r	p value
Age	0.209	0.173	0.034	0.825
Gender	− 0.014	0.929	− 0.057	0.712
Body mass index	0.144	0.351	− 0.026	0.868
Smoking index	0.099	0.524	− 0.028	0.855
PEF	− 0.309	0.041*	− 0.237	0.121
FEV_1_% predicted	− 0.393	0.008*	− 0.543	< 0.001*
FVC% predicted	− 0.264	0.083	− 0.424	0.004*
FEV_1_/FVC ratio	− 0.379	0.011*	− 0.433	0.003*

*PEF* peak expiratory flow, *FEV*_*1*_ forced expiratory volume in 1 s, *FVC* forced vital capacity

* Statistically significant difference

The multiple linear regression analysis with stepwise selection demonstrated that sestrin2 was independently associated with FEV_1_% predicted in asthmatics both during exacerbation and when controlled after the exacerbation (Table 3).

**Table 3:** Stepwise multiple regression model of sestrin2 levels in the asthma exacerbation and controlled asthma

	Asthma during exacerbation (adjusted R^2^ = 0.134)			Asthma when controlled (adjusted R^2^ = 0.279)		
	B (SE)	β	p value	B (SE)	β	p value
Constant	2.656 (0.334)		< 0.001*	3.096 (0.372)		< 0.001*
FEV_1_% predicted	− 0.013 (0.005)	− 0.393	0.008*	− 0.021 (0.005)	− 0.543	< 0.001*

Independent variables considered: age, body mass index, FEV_1_, peak expiratory flow (PEF), forced vital capacity (FVC), FEV_1_/FVC ratio, smoking index

*SE* standard error, *FEV*_*1*_ forced expiratory volume in 1 s

* Statistically significant difference

## Discussion

The present study demonstrated that the level of sestrin2 in patients with asthma was significantly higher than that of the healthy subjects. In addition, sestrin2 was negatively correlated with FEV_1_% predicted and FEV_1_/FVC ratio in asthmatics both during exacerbation and when controlled after the exacerbation. More importantly, multiple linear regression analysis showed that sestrin2 was independently associated with FEV_1_% predicted in asthmatics both during exacerbation and when controlled after the exacerbation.

It was reported that oxidative stress was involved in the pathogenesis of asthma [16, 17]. Airway oxidative stress results from a variety of causes, including excessive exposure to environmental pro-oxidants, airway infiltration of inflammatory cells, metabolic disorders, and decreased levels of antioxidants [18, 19]. Oxidative stress may be the result of inflammation and may also trigger and increase inflammation [17]. Sestrin2 has been found as a stress-responsive protein which was strong associated with excessive oxidative stress, hypoxia, and DNA damage [20]. In addition, hypoxia causes up-regulation of sestrin2 [10, 21, 22]. Hypoxia stimulates the mitochondria and increases ROS, results in hypoxic stress, and induces elevated level of sestrin2 [11]. When asthma is exacerbated, airflow is significantly limited, resulting in increased hypoxia, leading to oxidative stress [23]. When asthma is controlled, oxidative stress tends to decrease and may still exist [24]. Therefore, we speculated that the increase in sestrin2 of asthma patients might be related to the aggravation of hypoxia caused by airflow limitation.

FEV_1_% predicted is an important indicator of airway function, and the more FEV_1_% predicted decrease, the more severe the asthma is [1]. In this study, sestrin2 was negatively correlated with FEV_1_% predicted, and multiple regression analysis showed that sestrin2 was independently and negatively associated with FEV_1_% predicted, which meant that sestrin2 might be helpful to assess the FEV_1_% predicted and accordingly to assess the severity of asthma, especially for the patients whose FEV_1_% predicted could not be obtained because of the inability to perform lung function testing, such as patients who cannot cooperate, etc. It also meant that sestrin2 might be useful for guiding the treatment of asthma. In addition, studies had shown that sestrin2 could up-regulate autophagy through activating AMPK [25, 26]. Increasing studies suggested that the level of autophagy in airway smooth muscle cells was significantly increased in asthma [27, 28]. Therefore, the role of sestrin2 in asthma needs further researches to confirm.

It was reported that sestrin2 could inhibit the signal transduction of platelet-derived growth factor receptor-beta (PDGFRβ), an upstream regulator in the alveolar damage repair process [15]. Studies on asthma suggested that inhibition of PDGFRβ signaling, in the context of exposure to chronic air allergens, can lead to increased lung dysfunction and thickening of airway smooth muscles, resulting in airflow limitation [29]. The present study shows that sestrin2 is independently associated with FEV_1_% predicted which is an indicator of the severity of airflow limitation. Accordingly, we speculate that sestrin2 might be involved airway remodeling and airflow limitation in asthma.

Some limitations exist in this study. Firstly, this study is not a randomized research and the sample size is small. Secondly, we didn’t investigate the effects of related treatments on sestrin2, such as glucocorticoids, β2-adenosine receptor agonists, etc. In addition, another limitation was that time of discharge from hospital might not be the ideal time to capture the well controlled asthma data, though asthma control was assessed within 4 weeks after discharge, which resulted in the inclusion of some partly controlled asthma patients in this study. Nonetheless, our study shows that sestrin2 level significantly increases in asthma, which indicates that sestrin2 is involved in asthma.

## Conclusions

Sestrin2 is involved in asthma. Sestrin2 levels increase in asthmatics both during exacerbation and when controlled after the exacerbation. In addition, sestrin2 is independently associated with FEV_1_% predicted.

## Data Availability

The data that support the findings of this article are included within the article (Tables 1, 2, 3).

## Abbreviations

AMPK: AMP-dependent protein kinase
BMI: body mass index
CD93: cluster of differentiation 93
COPD: chronic obstructive pulmonary disease
FEV1: forced expiratory volume in 1 s
FVC: forced vital capacity
ICS: inhaled corticosteroids
GINA: global strategy for asthma management and prevention
LABA: long-acting beta2-agonist
LAMA: long-acting muscarinic antagonist
PDGFRβ: platelet-derived growth factor receptor-beta
SABA: short-acting beta2-agonist
